# Supplementary material for: Epidemiological Characteristics of Staphylococcus Aureus in Raw Goat Milk in Shaanxi Province, China
Source: Antibiotics (Basel). 2019 Sep 8;8(3):141. doi: 10.3390/antibiotics8030141 (PMC6783894; doi:10.3390/antibiotics8030141)
Supplement: Supplementary file 1 [file antibiotics-08-00141-s001.pdf]

Supplementary Materials:

Table 1. Primers used in this study.

Primer name	Primer sequence (5′-3′)	Fragment size (bp)	Annealing temperature (℃)	Reference
fnbA	GATACAAACCCAGGTGGTGG<br>TGTGCTTGACCATGCTCTTC	191	52	Zmantar et al., 2008
fnbB	ACGCTCAAGGCGACGGCAAAG<br>ACCTTCTGCATGACCTTCTGCACCT	197	62	Pereyra et al., 2016
clfB	TGCAAGTGCAGATTCCGAAAAAAAC<br>CCGTCGGTTGAGGTGTTTCATTTG	194	62	Klein et al., 2012
cna	AAAGCGTTGCCTAGTGGAGAC<br>AGTGCCTTCCCAAACCTTTT	192	54	Zmantar et al., 2008
bap	CCCTATATCGAAGGTGTAGAATTG<br>GCTGTTGAAGTTAATACTGTACCTGC	971	60	Darwish and Asfour, 2013
icaA	CCTAACTAACGAAAGGTAG<br>AAGATATAGCGATAAGTGC	1351	49	Wang et al., 2018
icaD	AAACGTAAGAGAGGTGG<br>GGCAATATGATCAAGATAC	381	49	Pereyra et al., 2016
sea	CCTTTGGAAACGGTTAAAACG<br>TCTGAACCTTCCCATCAAAAAC	127	55	Bayles, K, et al.,1998
seb	TCGCATCAAACTGACAAACG<br>GCAGGTACTCTATAAGTGCCTGC	477	53	Bayles, K, et al.,1998
sec	AGATTTAGCAAAGAAGTACAAAGATG<br>AAGGTGGACTTCTATCTTCACACTT	490	63	Sergeev, et al.,2004
sed	GAGGTGTCACTCCACACGAA<br>TGAAGGTGCTCTGTGGATAATG	349	57	Avanish K et al.,2009
see	ACCGATTGACCGAAGAAAAA<br>ATTGCCCTTGAGCATCAAAC	264	51	Avanish K et al.,2009
seg	AGAATTAGCTAACAATTATAAAGATAA AAAAG<br>TCAGTGAGTATTAAGAAATACTTCCAT	496	60	Sergeev, et al.,2004
seh	TGATTTAGCTCAGAAGTTTAAAAATAA AAATG<br>TTTCTTAGTATATAGATTTACATCAATAT	466	62	Sergeev, et al.,2004
sei	TGGAACAGGACAAGCTGAAA<br>TGTTTGCCATTAACCCAAAG	529	51	Avanish K et al.,2009
sej	ATGAAAAAAACAATATTTATACTGATTT TCTCCC	807	57	Sergeev, et al.,2004

	TCTACAGAACCAAAGGTAGACTTATTAATAC			
sek	ATGAATCTTATGATTTAATTTCAGAATCAA<br>ATTTATATCGTTTCTTTATAAGAAATATC	545	60	Sergeev, et al.,2004
sel	ATGAAAAAAAGATTATTATTTGTAATTGTTATTAC<br>ATCATCTTTTTGAAATTTCGACATCTAG	723	60	Sergeev, et al.,2004
sem	ATGAAAAGAATACTTATCATTGTTGTTTTATTG<br>CTTCAACTTTCGTCCTTATAAGATATTTC	258	60	Sergeev, et al.,2004
sen	ATAAAAAATATTAAAAAGCTTATGAGATTGTTC<br>ACTTAATCTTTATATAAAAATACATCAATATG	777	60	Sergeev, et al.,2004
seo	TATGTAGTGTAAACAATGCATATGCA<br>TCTATTGTTTTATTATCATTATAAATTTGCAAAT	685	58	Sergeev, et al.,2004
seq	GGAAAATACACTTTATATTCACAGTTTC<br>ATTTATTCAGTTTTCTCATATGAAATCTC	539	60	Sergeev, et al.,2004
ser	AGCGGTAATAGCAGAAAATG<br>TCTTGTACCGTAACCGTTTT	363	51	Holtfreter, et al., 2007
seu	AATGGCTCTAAAATTGATGG<br>ATTTGATTTCCATCATGCTC	215	49	Holtfreter, et al., 2007
tsst	AAGCCCTTTGTTGCTTGCG<br>ATCGAACTTTGGCCCATACTTT	447	53	Bayles, K, et al.,1998

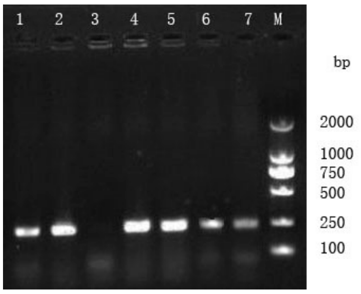

Figure 1. PCR amplification for the detection of *fnbA*, *fnbB* and *clfB* gene of *S. aureus* isolates. M: DL2000 marker; 1-3: PCR verification of *fnbA* gene with 191 bp, Line 1-3: different isolates from raw goat milk; 4-5: PCR verification of *fnbB* gene with 197 bp, Line 4-5: different isolates from raw goat milk; 6-7: PCR verification of *clfB* gene with 194 bp, Line 6-7: different isolates from raw goat milk.

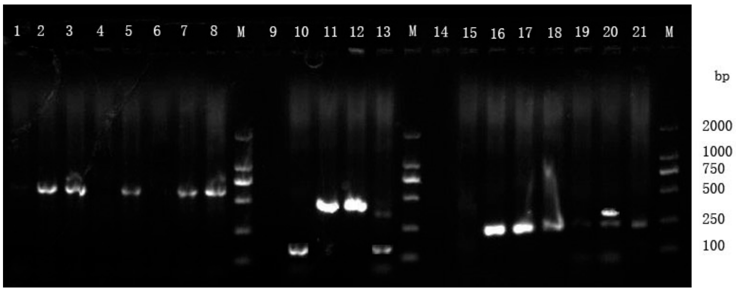

Figure 2. PCR amplification for the detection of *seo, sek, sei, seb, sea, cna, seu* and *see* genes of *S. aureus* isolates. M: DL2000 marker; 1-2: PCR verification of *seo* gene with 685 bp, Line 1-2: different isolates from raw goat milk; 3-4: PCR verification of *sek* gene with 545 bp, Line 3-4: different isolates from raw goat milk; 5-8: PCR verification of *sei* gene with 529 bp, Line 5-8: different isolates from raw goat; 9-10: PCR verification of *sea* gene with 127 bp, Line 9-10: different isolates from raw goat; 11-13: PCR verification of *seb* gene with 477 bp, Line 11-13: different isolates from raw goat; 14-15: PCR verification of *cna* gene with 192 bp, Line 14-15: different isolates from raw goat; 16-18: PCR verification of *seu* gene with 215 bp, Line 16-18: different isolates from raw goat; 19-20: PCR verification of *sek* gene with 264 bp, Line 19-20: different isolates from raw goat.

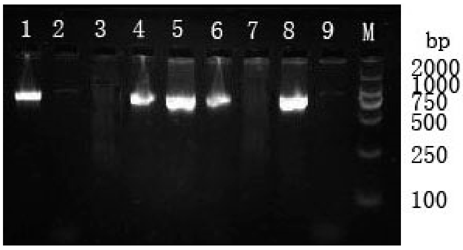

Figure 3. PCR amplification for the detection of *bap, sej* and *sen* genes of *S. aureus* isolates. M: DL2000 marker; 1-2: PCR verification of *bap* gene with 971 bp, Line 1-2: different isolates from raw goat; 3-4: PCR verification of *sej* gene with 807 bp, Line 3-4: different isolates from raw goat; 5-9: PCR verification of *bap* gene with 777 bp, Line 5-9: different isolates from raw goat.

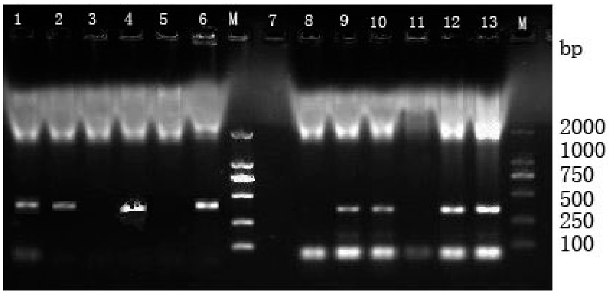

Figure 4. PCR amplification for the detection of *sec, seh, seg, tsst* and *ser* genes of *S. aureus* isolates. M: DL2000 marker;1-2: PCR verification of *sec* gene with 490 bp, Line 1-2: different isolates from raw goat; 3-4: PCR verification of *seh* gene with 466 bp, Line 3-4: different isolates from raw goat; 5-7: PCR verification of *seg* gene with 486 bp, Line 5-7: different isolates from raw goat; 8-10: PCR verification of *ser* gene with 363 bp, Line 11-12: different isolates from raw goat.

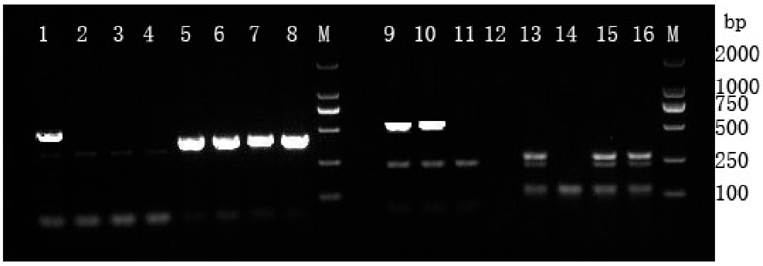

Figure 5. PCR amplification for the detection of *sel, sed, icaD, seq* and *sem* gene of *S. aureus* isolates. M: DL2000 marker; 1-2: PCR amplification of *sel* gene with 723 bp, Line 1-2: different isolates from raw goat; 3-5: PCR verification of *sed* gene with 349 bp, Line 3-5: different isolates from raw goat; 6-8: PCR verification of *icaD* gene with 381 bp, Line 6-8: different isolates from raw goat; 9-12: PCR verification of *seq* gene with 539 bp, Line 9-12: different isolates from raw goat; 13-16: PCR verification of *sem* gene with 258 bp, Line 13-16: different isolates from raw goat.
